# Berries vs. Disease: Revenge of the Phytochemicals

**DOI:** 10.3390/ph17010084

**Published:** 2024-01-09

**Authors:** Felipe F. Lamenza, Puja Upadhaya, Peyton Roth, Suvekshya Shrestha, Sushmitha Jagadeesha, Natalie Horn, Hasan Pracha, Steve Oghumu

**Affiliations:** 1 Department of Pathology, The Ohio State University Wexner Medical Center, Columbus, OH 43210, USA; lamenza.1@osu.edu (F.F.L.); upadhaya.1@osu.edu (P.U.); roth.633@buckeyemail.osu.edu (P.R.); shrestha.119@buckeyemail.osu.edu (S.S.); sushmithajagadeesha@osumc.edu (S.J.); horn.420@buckeyemail.osu.edu (N.H.); pracha.3@buckeyemail.osu.edu (H.P.); 2 Department of Microbiology, The Ohio State University, Columbus, OH 43210, USA

**Keywords:** black raspberries, phytochemicals, colorectal cancer, esophageal cancer, oral cancer, head and neck squamous cell carcinoma, anthocyanins

## Abstract

Secondary metabolites and phytochemicals in plant-based diets are known to possess properties that inhibit the development of several diseases including a variety of cancers of the aerodigestive tract. Berries are currently of high interest to researchers due to their high dietary source of phytochemicals. Black raspberries (BRB), *Rubus occidentalis*, are of special interest due to their rich and diverse composition of phytochemicals. In this review, we present the most up-to-date preclinical and clinical data involving berries and their phytochemicals in the chemoprevention of a variety of cancers and diseases. BRBs possess a variety of health benefits including anti-proliferative properties, anti-inflammatory activity, activation of pro-cell-death pathways, modulation of the immune response, microbiome modulation, reduction in oxidative stress, and many more. However, little has been done in both preclinical and clinical settings on the effects of BRB administration in combination with other cancer therapies currently available for patients. With the high potential for BRBs as chemopreventive agents, there is a need to investigate their potential in combination with other treatments to improve therapeutic efficacy.

## 1. Introduction

As arguably one of the most gripping health issues of the 21st century, cancer is a group of diseases in which altered cells divide uncontrollably and damage healthy tissues in the body [1]. In many developed countries, cancers have surpassed cardiovascular diseases as the leading cause of death, and in some cases, account for twice as many deaths as cardiovascular diseases [2,3]. In the United States, it is estimated that roughly 1.9 million new cancer cases will be diagnosed, with a projection of approximately 600,000 deaths, in 2023 [1]. In the context of global epidemiological data, it is predicted that approximately 14.1 million new cancer cases and 8.2 million cancer deaths occur each year, with the mortality rate varying depending on the cancer type, subtype, patient demographic characteristics and medical parameters of a particular country or region [4]. These findings suggest that there is an urgent need for a better understanding of disease development and progression, and novel therapeutic approaches.

Diet can have positive or negative effects on health. In cancer, diet may either inhibit or promote their development. Past studies have specifically identified tumor-promoting dietary constituents, such as diets that contain high levels of refined sugar, low levels of dietary fiber, processed red meat [5], a high ratio of omega-6 fatty acids to omega-3 fatty acids, high levels of sodium intake, and increased rates alcohol consumption [5]. On the other hand, certain dietary patterns can inhibit the development and proliferation of tumors while promoting overall well-being. For instance, the Mediterranean diet stands out for its emphasis on abundant consumption of fruits, vegetables, whole grains, legumes, olive oil and other unsaturated fats, and lean protein sources like fish and chicken. Notably, this diet avoids excessive intake of refined sugar, red meat, and saturated fats, all of which are potential cancer-promoting agents [6]. Recent studies have found that the Mediterranean diet prevents tumor development and lowers the mortality and recurrence rates in many cancer types [6].

Epidemiological evidence demonstrates that individuals with increased consumption of plant-based diets are associated with a decreased risk of cancer development, especially cancers of the aerodigestive tract [7]. Many of the health benefits from plant-based diets stem from their phytochemical or secondary metabolites composition. These provide unique and novel structural diversity which present opportunities for novel drug candidates [8,9,10]. Berries have especially gained traction due to their high abundance of these phytochemicals [11] which demonstrate anti-cancer properties [12,13], such as antioxidant, immuno-modulatory, and anti-inflammatory properties [12,13]. The rich composition of vitamins, minerals, fibers, polyphenols, and other bioactive components are evidently the source of the anti-cancer properties of berries [14].

**Figure 1:**
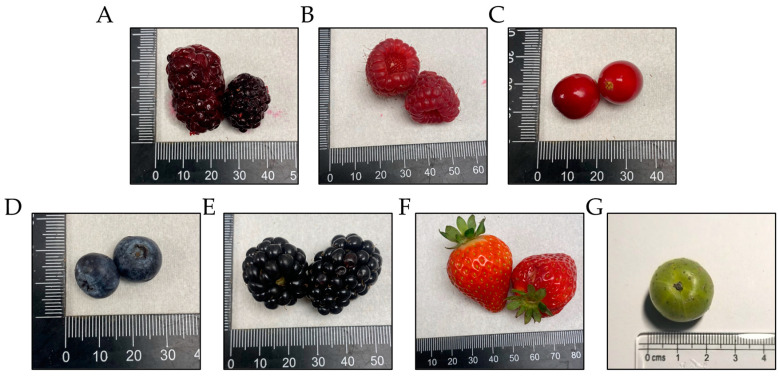
Representative images of (**A**) black raspberries, (**B**) raspberries, (**C**) cranberries, (**D**) blueberries, (**E**) blackberries, (**F**) strawberries, and (**G**) Indian gooseberry.

Berries that have been found to contain high levels of bioactive components typically include black raspberries, raspberries, cranberries, blueberries, blackberries, strawberries, and Indian gooseberries (Figure 1A–G) [15]. Black raspberries (BRB; Rubus occidentalis) are especially rich in phytochemicals such as phytosterols (β-sitosterol), and a variety of polyphenols such as ellagic acid, ferulic acid, ellagitannins, anthocyanins, protocatechuic acid, and bioflavonoids (quercetin), among others which contribute to a positive impact on health [16,17,18,19]. In this review, we will focus on how BRBs and their phytochemicals have been used in dietary studies for chemoprevention of a variety of cancers. We will also highlight areas where further studies on how BRB phytochemicals may potentially be exploited in combination with other cancer treatments to increase their efficacy.

## 2. Methodology

A comprehensive literature search was undertaken to review preclinical and clinical data on the role of berries and their phytochemicals in the chemoprevention of various cancers and diseases. This search encompassed articles published from January 1977 to August 2023, utilizing scientific electronic databases such as SCiFinder, PubMed, Scopus, ScienceDirect, and Google Scholar library. For PubMed searches, medical subject heading (MeSH) terms were employed. The inclusion criteria for the papers in this review were strictly limited to peer-reviewed studies that demonstrated clear research methodologies, adequate sample sizes, and findings of statistical significance. This approach was assumed to guarantee that the review was methodical, replicable, and concentrated on relevant, high-caliber research.

## 3. Black Raspberries and Cancer

### 3.1. Head and Neck Cancer

**Figure 2:**
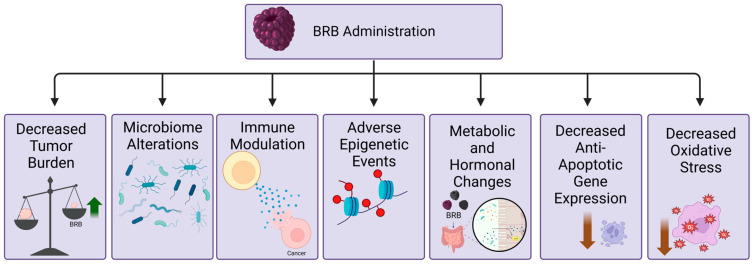
Schematic representation of the effects of BRB administration in cancers of the aerodigestive tract (created with BioRender.com accessed 29 December 2023). BRB-mediated decrease in tumor burden can be attributed to alterations in the microbiome, modulation of the immune response, changes in epigenetic events, metabolic and hormonal pathways, decreased expression of anti-apoptotic genes, and decreased oxidative stress.

Most malignancies from the head and neck region are derived from the mucosal epithelium in the oral cavity, pharynx, and larynx, which are known collectively as head and neck squamous cells carcinomas (HNSCC) [20]. In the past few years, several research studies have been conducted to assess the potential of using BRB independently or in conjunction with other treatments for addressing oral cancer. In a study conducted by Chen et al., it was found that in mice exposed to dibenzo pyrene (DBP), an environmental pollutant found in polyaromatic hydrocarbons and a constituent of tobacco smoke, dietary administration of 5% significantly decreased tumor incidence (70% to 46.7%). BRB administration also resulted in a significant reduction in the levels of DBP-DNA adducts within the oral cavities of the mice [21]. Other mechanisms associated with BRB-mediated chemoprevention of HNSCC in this model include increased p120ctn expression [22]. In a different animal model of HNSCC, 7,12-dimethylbenz(a)anthracene (DMBA), induced hamster cheek pouch (HCPs), the preclinical efficacy of the topical application of BRB on chemoprevention of oral cancer was demonstrated by reduced tumor multiplicity, tumor incidence, and proliferation rate. Short-term topical delivery of BRB significantly up-regulated gene expression of retinoblastoma, a canonical tumor suppressor gene that is inactivated during HNSCC progression in high-at-risk mucosa of DMBA-induced HCPs [23]. These studies were corroborated in another HCP HNSCC model using 5% and 10% lyophilized BRB as dietary chemopreventive agents. Interestingly, 5% BRB was more effective at inhibiting HNSCC tumor development than 10% BRB. (Figure 2) These results provide support for earlier studies demonstrating the chemopreventive potential of BRB and, importantly, establish that a dietary intake of BRB can inhibit tumor formation in the oral cavity [24].

Our group has previously demonstrated the effect of BRB on 4NQO-induced HNSCC in F344 rats. Like the HCP model, dietary administration of 5% and 10% BRB reduced the incidence and multiplicity of oral lesions by 39.3% and 28.6%, respectively. The inhibition of oral lesion by BRB was associated with reduced gene expression of anti-apoptotic and cell cycle associated markers (Aurka, Birc5, Ccna1, and Ccna2), and protein expression of Ki-67 in tongue epithelial tissues. The polyphenol ellagic acid, a phytochemical in BRB, showed similar effects [25]. Novel mechanisms of BRB chemoprevention have been identified using experimental animal models. A recent report using 4NQO-induced HNSCC in C57Bl/6 mice and F344 rats showed BRB mediated the modulation of HSD11B2, an enzyme which mediates the conversion of the active glucocorticoid cortisol into inactive cortisone. These findings demonstrated that BRB extract upregulated the HSD11B2 gene and protein expression in HNSCC cells in vitro and in vivo, resulting in a concurrent reduction in the levels of active glucocorticoids in HNSCC-induced mouse tongues (Figure 2) [26].

The immunomodulatory effect of BRB in the HNSCC tumor microenvironment has been explored in our laboratory. Our findings revealed that dietary BRB administration can inhibit the recruitment of regulatory T cells while simultaneously enhancing cytotoxic CD8 T cell activity within the tumor microenvironment. This enhanced activity was characterized by an increased production of granzyme B in the tumor site during BRB-mediated chemoprevention of HNSCC [12]. These results were further corroborated in a novel genetically modified mouse model of HNSCC involving intralingual tamoxifen injection in mouse with a conditional deletion of Tgfβr1 and Pten which resulted in HNSCC tumors that closely resembled clinical HNSCC in histology, molecular profile, and lymph node metastasis. Using this model, BRB administration over a 5-week period led to several noteworthy outcomes, including a reduction in tumor growth, an increased influx of anti-tumoral T cells into the tumor microenvironment, and the augmentation of anti-tumoral cytotoxic CD8+ T cell activity, characterized by increased expression of granzyme B and perforin (Figure 2) [27]. Collectively, the studies provide evidence for BRB-mediated promotion of anti-tumoral immune responses, mediated by improved anti-tumoral T cell infiltration, which opens the potential of combinatorial approaches with checkpoint inhibitors for improved HNSCC treatment outcomes.

Several studies have reported on the potential efficacy of BRB formulations in the chemoprevention of HNSCC in clinical patients. A 6-week trial was performed utilizing a mucoadhesive gel formulation of 10% freeze-dried BRB [28,29,30]. Patients with oral pre malignancies applied the gel four times daily, delivering 0.5 g of BRB in each application. Patients were biopsied before and after the 6-week trial period, along with normal tissue for comparison [29]. No patients within the study reported adverse events from the gel application. BRB gel treatment resulted in histological regression in a subset of patients and a statistically significant reduction in the loss of heterozygosity [29]. This study was further expanded in the same cohort with the goal to explore alteration in growth factors, proinflammatory and angiogenic enzymes, gene expression profiles, and micro vessel density [31]. The BRB gel showed greater suppression of genes associated with RNA processing, growth factor recycling, and inhibition of apoptosis. Furthermore, COX-2 levels were reduced along with micro vessel density [31]. These studies are very informative on potential translational applications of BRB formulations in HNSCC chemoprevention. Interestingly, the authors noted that a subpopulation of patients appeared more responsive to the treatment [31]. Underlying biological and molecular mechanisms that distinguish responders from non-responders to BRB chemoprevention of HNSCC will be crucial in determining clinical application. Further expansion of this study to a multi-centered placebo-controlled trial involving forty patients over a 3-month period [28] was performed. A total of 22 patients received the 10% BRB gel treatment, while 18 patients received the placebo gel. Application of the gels occurred four times daily with a total of 0.5 g of BRB being administered with each application to the oral premalignant lesion. The BRB group saw significant reductions in lesion size, histological grade, and loss of heterozygosity compared to the placebo group [28]. However, variation in responsiveness to the BRB treatment was observed among patients, as measured by the different levels of BRB metabolic and keratinocyte differentiation enzymes in patient lesions [28]. In another clinical trial, patients with confirmed oral squamous cell carcinomas (OSCC) were administered three dissolvable slow-release oral troches daily containing 4.3 g freeze-dried BRB powder until resection surgery [32]. Following BRB administration, pro-survival genes AURKA, BIRC5, and EGFR and proinflammatory genes NFKB1 and PTGS2 were significantly reduced. Furthermore, the phytochemicals cyanidin-3-rutinoside and cyanidin-3-xylosylrutinoside were detected in all OSCC patient tissues (Figure 2) [32]. These studies demonstrate the ability of BRB to modulate the transcriptional profile of HNSCC cells in a manner that supports a chemopreventive role, and builds on the findings that local delivery of BRB treatment to the target site results in better and more consistent absorption in HNSCC patients, which suggests an effective strategy for management of these cancers [33].

### 3.2. Esophageal Cancer

Esophageal cancers (EC) are derived from the cells that line the inside of the esophagus, with most being adenocarcinomas and squamous cell carcinomas [34]. Its subtle early symptoms and aggressive nature make it a challenging cancer to treat, with only a 15–20% five-year survival rate [35]. As there is an ongoing search for innovative ways to prevent and treat EC, the potential role of BRBs has garnered attention for its promising properties [36]. In a recent study by Shi et al., N-nitrosomethylbenzylamine (NMBA)-induced rats were used to assess the effect of BRB on oxidative stress and its related oncogenic signaling pathways such as the NF-kB/MAPK to further understand the underlying mechanisms of the anti-cancer action of BRB. Tumor incidence and tumor multiplicity in rats fed 5% BRB was significantly reduced as compared to those fed a control diet. In addition, the mRNA expression of SOD2 (an essential antioxidant defense against oxidative stress) and GPx (glutathione peroxidase, an antioxidant enzyme that scavenges free radicals to prevent lipid peroxidation as well as maintain redox balance to prevent damage to DNA, proteins, and lipid membranes) [37,38], were assessed. While SOD2 and Gpx expression were decreased in NMBA-induced rats leading to oxidative stress, NMBA-induced rats fed a BRB diet demonstrated increased SOD2 and GPx gene expression. BRB was also observed to significantly suppress the phosphorylation of the oncogenic signaling NF-κB and MAPK pathways. Overall, this study showed that BRB contributes to the alleviation of EC incidence as well as tumor multiplicity by reversing oxidative stress and suppressing NF-kB/MAPK pathways, suggesting that this could be a potential mechanism of EC chemoprevention by BRB (Figure 2) [39]. Similar results were found in other studies evaluating the effects of dietary BRB on experimentally induced EC, with additional mechanisms associated with pro-inflammatory cytokines modulation (including CCL2, S100A8, and IL-19), prevention of aberrant DNA methylation, which is a common occurrence in the development of EC [40], and reduced gene expression of DNA methyltransferases, Dnmt1 and Dnmt3b, in both dysplastic lesions and in papilloma of the esophagus [41].

The bioactive phytochemicals in BRB have been further explored in EC chemoprevention [42]. Peiffer et al. explored the role of BRB and its component anthocyanins (AC) and protocatechuic acid (PCA) in inhibiting the development of EC in rats induced by NMBA. Rats were fed with diets containing 6.1% BRB powder, an AC-rich fraction of BRBs (3.8 μmol/g), or 500 ppm PCA. All three diets had similar effects on cytokine production in the plasma and esophagus with decreased production of pro-inflammatory cytokine IL1β and increased expression of the anti-inflammatory cytokine IL-10. An increased expression of IL-12, which is known to activate cytotoxic NK cells and CD8+ T cells, was also observed in all dietary administrations. Downregulation of pro-inflammatory cytokines, upregulation of anti-inflammatory cytokines and alteration of the immune cell infiltration into tumor tissues were demonstrated to be mechanisms associated with BRB-, AC-, and PCA-mediated chemoprevention of EC (Figure 2) [43].

In addition to animal studies that employed NMBA-induced rat models, a few in vitro studies have explored the mechanistic role of BRB as well as its individual phytochemical constituents in the prevention of esophageal carcinogenesis. One such study evaluated an ethanol (EtOH) extract of BRB and the two component anthocyanins (cyanidin-3-O-glucoside and cyanidin-3-O-rutinoside) in BRB for their effects on growth, apoptosis, and gene expression in rat esophageal epithelial cell lines RE-149 and RE-149 DHD. A dose-dependent growth inhibition of RE-149 DHD cells by BRB extract was observed. An amount of 100 µg/mL was the most effective inhibitory concentration of the extract, resulting in a 45% and 16% inhibition in the growth of RE-149 DHD and RE-149 cells, respectively. However, the growth inhibition was significant only for the RE-149 DHD cells. Similar to BRB extract, cyanidin-3-O-glucoside and cyanidin-3-O-rutinoside, were seen to selectively cause significant growth inhibition and induction of apoptosis in a highly tumorigenic cell line RE-149 DHD but not in a weakly tumorigenic line RE-149. Mechanistically, increased Caspase 3 and Caspase 7 expression were associated with effects of BRB and two component anthocyanins on treated cells. However, a significant reduction in COX-2 and i-NOS expression was observed only with cyanidin-3-O-glucoside or cyanidin-3-O-rutinoside but not BRB extract. Taken together, this study revealed that the EtOH BRB extract and two component anthocyanins inhibit the growth and apoptosis of highly tumorigenic rat esophageal epithelial cells in vitro by affecting the gene expressions of Caspase 3 and 7 as well as COX-2 and i-NOS (Figure 2) [44]. Other associated mechanisms of BRB phytochemical-mediated inhibition of EC as revealed by in vitro studies include the suppression of human β-defensin 2 (HBD-2), a protein expressed in the esophagus in response to stress or infectious agents that possesses an oncogenic role in the initiation and progression of esophageal SCC. These effects were observed in cyanidin-3-glucoside and cyanidin-3-rutinoside treatment of the esophageal SCC cell line KYSE-150, in a dose-dependent manner, which was similar to treatment with cancer drugs PBIT and celecoxib [45].

The studies described above show that BRB and its components are effective in alleviating esophageal carcinogenesis by reducing tumor incidence as well as multiplicity by modulating several inflammatory, oxidative stress, and oncogenic pathways. Moreover, it is demonstrated that BRB has a rich profile of diverse phytochemicals, which allows for suppression of carcinogenesis through multiple mechanisms of action which may be synergetic and interactive. This suggests that there are unestablished mechanisms by which BRB and its components interact with these pathways, which is an interesting area of research that can be further explored. Moreover, most of the studies exhibited BRB’s chemopreventive effects on EC in N-nitrosomethylbenzylamine (NMBA)-induced rats. Tumors induced by the nitrosamine carcinogen N-nitrosomethylbenzylamine (NMBA) have been used extensively for studies of the molecular mechanisms involved in the development of esophageal SCC as well as for the examination of the chemopreventive effects of BRB. It is also noteworthy, as suggested by the studies discussed above, that 5% BRB appears to be an effective BRB dose for EC chemoprevention. The opportunity to enhance BRB efficacy via combinatorial approaches remains a largely unexplored area for additional investigation.

Several studies have further assessed the chemopreventive effect of BRB on clinical EC patients. A 6-month pilot study was conducted to assess the long-term tolerability and efficacy of food-based chemopreventive approaches by investigating whether BRBs can modulate different signaling cascades associated with the development of EC [46,47]. Twenty patients were given lyophilized or freeze-dried BRB powder mixed with water at 32 and 45 g once a day, which is equivalent to concentrations of BRB powder that were demonstrated to inhibit EC in animal models [48,49,50]. The study showed that the ellagitannin metabolite, Urolithin A-glucuronide, significantly increased following BRB treatment for 12 and 26 weeks compared to baseline, which indicates that this is a good biomarker for treatment compliance. Furthermore, BRBs significantly decreased urinary excretion of 8-PGF2α, which is considered a reliable and relatively stable marker for oxidative stress and an indicator of lipid peroxidation (Figure 2). However, Ki-67 levels, a marker for cancer cell proliferation, and histopathology grade did not change with the treatment [46,47]. Additional studies will be needed to determine whether improved efficacy can be achieved with combinatorial approaches with BRBs for clinical EC patients.

### 3.3. Colorectal Cancer

Colorectal cancers (CRC) originate from cells in the inner lining of the colon or rectum that start off as growths known as polyps [51]. Most tend to be adenocarcinomas derived from mucus-producing cells [51]. In vitro studies demonstrate an important role for BRBs in inhibiting CRC growth, and several mechanisms have been identified. One such study using human CRC cell lines SW480 and Caco2, demonstrated a growth inhibitory effect of a BRB extract, with mechanisms associated with the promotion of histone acetylation, upregulation of p65, and downregulation of IκB, pathways which are known to be important in CRC progression [52,53,54]. Similar inhibitory effects of BRB on cancer cell proliferation, migration, and colony formation in vitro were observed in HCT-116 and LoVo CRC cell lines [55].

In vivo studies using a mouse model of CRC with azoxymethane (AOM-induced CRC model) corroborates these in vitro results [56]. This study demonstrated that a BRB diet (4.1 g/kg) significantly reduced tumor multiplicity, emphasizing BRB’s role in preventing the progression of CRC. In line with previous in vitro experiments, these in vivo studies demonstrate BRB-mediated reduction in the anti-apoptotic protein Bcl-2 and increased expression of the apoptotic protein, Bax (Figure 2) [53]. Similarly, Chen et al. observed a significant reduction in tumor multiplicity in a chemically induced CRC (AOM/DSS) mouse model after dietary administration of 5% and 10% BRB [55].

BRB has also been shown to modulate antitumor immunity in CRC. Innate immune cells such as natural killer (NK) cells play a significant role in mitigating CRC development and progression. A recent study investigated underlying mechanisms of NK cell effects on CRC progression, and the potential role BRB extract plays in modulating this innate immune population during CRC chemoprevention [57]. BRB was shown to enhance NK cell infiltration and suppress CRC development. Studies on clinical CRC patients further demonstrate the efficacy of BRB phytochemicals in enhancing NK cell cytotoxicity [58]. Further evidence for BRB-mediated immunomodulation in CRC is demonstrated by other studies investigating the potential of BRBs to modulate inflammation and cytokine production during CRC [59]. Growing evidence has shown that inflammation and cytokines can promote carcinogenesis including processes in the development of CRC [60]. Twenty-four patients with CRC, who had not received prior treatment, consumed 20 g of freeze-dried BRB powder three times daily for three weeks until surgical resection. Plasma concentration of granulocyte macrophage colony-stimulating factor (GM-CSF) and IL-8 were significantly reduced with the BRB diet which was associated with the induction of apoptosis and reduced proliferation in colorectal tissues [59].

Epigenetic modification of tumor suppressor genes has been found to be an associated mechanism of BRB chemoprevention in clinical CRC patients. Wang et al. sought to evaluate the effects BRBs have on biomarkers of tumor development in human CRC, specifically focusing on methylation patterns of relevant tumor suppressor genes, cell proliferation, apoptosis, angiogenesis, and Wnt pathway genes [61]. In this study of 20 enrolled patients, BRB powder was administered orally (60 g/day for 1 to 9 weeks), a concentration equivalent to a rodent diet of about 7% BRB powder, which was confirmed to have chemopreventive properties in the rat colon [14,62]. Patients enrolled in this study were not currently receiving chemotherapy or radiation therapy. Immunohistochemical data from biomarkers Ki-67, TUNEL, β-catenin, CD105, and DNMT1 indicated that they were modulated protectively in the tissues of all patients. However, positive DNA methylation modulation only occurred in patients that were treated with BRBs for an average of 4 weeks, suggesting that BRB treatment required a relatively long period of administration to be effective [61]. Studies have shown that DNA methyltransferase (DNMTs) are overly expressed in several tumor types including CRC, and knockout experiments with cell lines have demonstrated increased expression of tumor suppressor genes such as p16 (Figure 2) [63,64]. Therefore, inhibition of DNMTs may be a promising target for chemoprevention of CRC [65]. This study provided evidence of the ability of BRBs to demethylate tumor suppressor genes and to modulate other biomarkers of tumor development in human CRC.

Metabolomic analysis has further revealed additional mechanisms associated with BRB-mediated chemoprevention of CRC. One such study by Pan, P. et al. [66] investigated BRB-mediated metabolite changes from 28 CRC patients who were given 60 g of BRB powder daily for 1 to 9 weeks, following the same administration protocol as Wang et al. [61]. Patients in this study were also not currently receiving chemotherapy or radiation therapy. Non-targeted metabolic analysis uncovered over 400 annotated metabolites, with 34 and 16 metabolites significantly changed by BRB in the urine and plasma, respectively. Increased levels of 4-methylcatechol sulfate were observed in both post-BRB urine and post-BRB plasma, which were correlated with higher levels of apoptosis in post-BRB tumors. Furthermore, polyphenols derived from BRB were absorbed and metabolized to various benzoate species, which were found to be increased in post-BRB patients. These were correlated with enhanced levels of amino acid metabolites [66]. This study suggests BRBs induce significant metabolic changes in CRC patients, and BRB-mediated regulation of metabolic pathways may be beneficial against CRC.

The chemopreventive properties of BRB have also been evaluated in patients with precancerous lesions. A pilot study was conducted on patients with familial adenomatous polyposis (FAP), an inherited disorder characterized by early onset of colonic polyposis which can progress into colon and rectum cancer [67,68]. Non-steroidal anti-inflammatory drugs (NSAIDs) have been found to regress FAP-associated polyps, but non-toxic alternatives treatments are being investigated [69,70,71]. The 14 patients enrolled in this study either received a maltodextrin placebo powder three times daily, combined with two BRB rectal suppositories administered at night, or consumed freeze-dried BRB powder (20 g) mixed with water three times daily with the two BRB rectal suppositories administered at night [72]. Before treatment initiation and at the 36-week mark of the study, patients underwent sigmoidoscopy to record polyp number, size, and burden. The study concluded that the BRB suppositories decrease the tumor burden of patients with no additional benefits from the oral consumption of BRB. However, the authors noted that a sample size greater than seven is likely needed to properly assess whether combined oral consumption of BRB with suppository treatment offers an increase in efficacy compared to suppository alone. Like other studies, further analysis demonstrated that BRBs also decreased the expression of Ki-67 and DNMT1, while increasing the expression of P16 [72]. Overall, the data collected from BRB human colorectal studies have been encouraging, with minimal toxicity and adverse effects.

Combinatorial treatment approaches with BRB have the potential to enhance anti-tumor efficacy against CRC and to overcome potential problems associated with treatment resistance. A recent study investigated the combined effect of BRB anthocyanins and 5- fluorouracil (5-FU) or celecoxib (Cel) on CRC, using SW480 and Caco2 cells [73]. While BRB anthocyanins alone did not affect cell proliferation, a significant reduction in cell proliferation was observed in combination treatments with BRB + 5-Fu or BRB + Cel, compared to 5-Fu or Cel alone. These findings were consistent for both cell lines (SW480 and Caco2); hence, they are suggestive of a synergistic anti-tumor effect of BRB + 5-Fu/Cel. These in vitro findings were reassessed in an in vivo setting using the AOM-induced mouse CRC model. In this study, 5-Fu was injected intraperitoneally at 40 mg/kg while BRB was administered through diet. Interestingly, the 5-Fu + BRB group showed reduced tumor multiplicity compared to 5-Fu alone, demonstrating an important role for BRB in combinatorial approaches for CRC chemoprevention and treatment. Further, 5-Fu + BRB administration maintained intestinal integrity, reduced disruption, and fewer inflammatory cells in AOM-induced mice. AKT signaling was also shown to play a role in the observed effects. p-AKT, HIF1-α, and MDR1, as well as EZH2 and H3K27me3 were shown to be significantly downregulated in 5-Fu + BRB compared with 5-Fu treatment alone [73]. These findings suggest a promising role for BRB in potentiating the anticancer efficacy of chemotherapeutic drugs.

## 4. Black Raspberries and Other Diseases

### 4.1. BRBs and Allergic Inflammation

Allergic inflammation arises from a distinct set of cellular and humoral reactions, culminating in the initiation of both the innate and adaptive immune systems. The dietary impacts of BRB have been observed in allergic conditions such as contact hypersensitivity (CHS). Our in vivo findings indicate that dietary administration of BRB extract and its anthocyanin metabolite, protocatechuic acid (PCA), can alleviate the pathology of contact hypersensitivity [74]. Mice on BRB- or PCA-supplemented diets had reduced contact allergen-induced ear swelling and less accumulation of activated DCs in the spleen after exposure to di-nitro fluorobenzene (DNFB). In vitro, BRB extract decreased DC maturation, Cd80 expression, and IL-12 secretion, while PCA reduced IL-12 levels. Dietary BRB and PCA led to varying decreases in IL-12-related CHS-mediated effector mechanisms, including IFN-γ, IL-2, and IL-17 production by T cells [74,75]. BRB fractions and a mixture of these cyanidins present in BRBs were effective in reducing lipopolysaccharide (LPS)-induced iNOS expression and other inflammatory markers such as tumor necrosis factor (TNF)-α, IL-6, and IL-1β. These effects were achieved by inhibiting the phosphorylation of mitogen-activated protein kinases (MAPKs) and STAT3 in murine macrophage RAW264.7 cells [76]. The anti-inflammatory properties of BRB phytochemicals contribute to their wide application in mitigating the pathology of diseases characterized by chronic inflammation.

### 4.2. BRBs and Cardiovascular Disease

BRBs have gained recognition for promoting cardiovascular well-being, evidenced by their capacity to lower levels of blood trimethylamine-N-oxide (TMAO), a metabolite produced by gut bacteria [77]. This metabolite has been linked to cardiovascular disease and was investigated in a study conducted using Sprague-Dawley rats, which were subjected to a high-fat diet supplemented with TMAO. Notably, dietary intake of TMAO resulted in elevated serum LDL cholesterol, whereas the consumption of BRB extract contributed to a reduction in this cholesterol biomarker [77]. In a separate study, it was demonstrated that BRB extract possesses the capability to induce alterations in the gut bacterial community. Additionally, BRB can modulate bile acids and regulate gene expression patterns, all of which collectively contribute to the potential of reducing cholesterol levels [78]. An investigation focusing on vascular senescence, a process associated with the advancement of cardiovascular diseases, revealed that polyphenol extracts from BRB displayed efficacy in diminishing Angiotensin II-induced senescence. This effect was achieved by increasing the cellular antioxidant capabilities, notably by elevating the expression of key antioxidant enzymes, including superoxide dismutase (SOD) 1, SOD2, and glutathione peroxidase [79].

BRB extract was utilized to investigate its potential anti-inflammatory and metabolic modulatory effects during choline-induced inflammation on rats fed a high-fat diet [80]. Various epidemiological studies have revealed a connection between choline and cardiovascular diseases, diabetes, and renal diseases, specifically focusing on vascular inflammation, endothelial dysfunction, and cholesterol homeostasis [81,82,83,84,85,86,87]. The study showed that a consistent intake of BRB extract was able to lower the levels of trimethylamine-N-oxide (TMAO) cecal and trimethylamine (TMA) serum, products of metabolized choline, which indicates an improvement in the serum lipid profile in diet-induced hypercholesterolemia in rats [88]. This suggests that BRBs may act as a prebiotic in the human gut and an agent to alleviate hepatic inflammation [80]. However, as the authors noted, further studies including microbiome analysis are needed to understand these observations.

### 4.3. BRBs and Hepatic Inflammation

Alcohol use disorder (AUD) is a significant public health concern on a global scale [89]. Excessive and long-term consumption of alcohol can lead to adverse effects in the digestive, urinary, and circulatory system [90]. Alcohol abuse may lead to alcoholic liver disorders (ALD) which include alcoholic hepatitis, fatty liver, liver fibrosis, and cirrhosis [91,92]. Anthocyanins from BRBs have been tested to determine effects on protection against alcohol liver diseases and hepatic inflammation. Xiao T et al. demonstrated the potential preventative role of BRB on ALD. Histopathological observations in sub-acute ALD mice administered BRBs displayed reversal of liver damage. Additional evidence for regulation of inflammation was found through decreased expression levels of NF-kb and TGF-b after treatment with BRBs in both acute and subacute treatment groups. The results demonstrate that BRBs possess preventative effects on ALD [93].

### 4.4. BRBs and Myelodysplastic Syndromes

BRBs have been used in a pilot clinical study to investigate their potential to treat myelodysplastic syndromes (MDS) and myelodysplastic/myeloproliferative neoplasms (MDS/MPN). These are bone marrow disorders characterized by cytopenia and eventual progression into acute myeloid leukemias [94]. Stem cell transplantation remains the only cure, but transfusions and growth factor therapies are also used for low-risk MDS [95]. Two FDA-approved drugs, azacytidine and decitabine, are used in high-risk MDS patients who can tolerate them; however, these drugs are associated with significant cytopenia and gastrointestinal toxicity [96]. A group of researchers who previously demonstrated the hypomethylating effects of BRBs in patients with colon cancer and familial adenomatous polyposis explored if BRB displayed similar hypomethylating effects as in conventional MDS treatment agents [61,72]. The trial demonstrated that a daily dose of 50 g of BRB powder mixed in water taken over the course of three months by patients with MDS and MDS/MPN lead to regulation of leukocytes and T-cell differentiation along with increased white blood cell counts. These observations were attributed to both a hypomethylation of intergenic regions and a hypermethylation of intragenic and intergenic regions. These effects also manifested without significant side effects or adverse effects in study patients which demonstrates the desired safety profile of BRB intervention [97]. The precise impact of the observed hypermethylation events will require further investigation.

### 4.5. BRBs and the Microbiome

The microbiome plays an important role in nutrient breakdown and synthesis, providing an antimicrobial barrier against potential pathogens, providing signals for immunomodulation, and assisting in the retention of structural components where they reside [98,99]. Within the gastrointestinal tract, there is mounting evidence demonstrating the integral role that a healthy microbiome plays in the prevention and detection of several diseases [100,101]. Major aberrations within the gastrointestinal microbiome have been associated with luminal diseases like inflammatory bowel disease (IBD) and irritable bowel syndrome, metabolic diseases like obesity and diabetes, allergic conditions, and neurodevelopmental illnesses [102,103,104,105]. Evidently, the microbiome plays a major role in the maintenance of healthy tissue and affects disease progression and prognosis [106,107]. Even as an understanding of relations between disease outcomes and gut microbial patterns have begun to emerge, the associations between host and gut bacteria metabolites are not well documented. Specialized metabolites within the gut microbiome have now been demonstrated as contributing to alterations in normal host physiology [105,108,109]. Metabolic products from bacteria which bind to the aryl hydrocarbon receptor (AHR) have been shown to affect immune cells and the mucosal lining [110,111]. Conditions such as Crohn’s disease and ulcerative colitis involve a complex disarray of inflammation within the digestive tract from abnormal gut bacteria and host immune responses. AHR expression has been found to be diminished within individuals suffering from IBD; however, diets supplemented with BRB increased fecal AHR activity in experimental studies [112,113]. These results display the potential that a diet supplemented with BRB may have on the modulation of key metabolites reducing intestinal inflammation associated with IBD. BRB phytochemicals and other constituents such as vitamins, calcium, and fiber [16] are metabolized by host microbes, generating secondary metabolites that contain antioxidant, antiproliferative, and pro apoptotic properties [114,115]. It has been shown that BRBs are able to promote the production of short-chain fatty acids utilizing gut microbe fermentation [116]. Furthermore, BRB consumption has been associated with the promotion of beneficial gut bacteria growth, like *Akkermansia muciniphila*, *Bifidobacterium* species, and Lactobacillus species, while inhibiting the growth of pathogenic strains and species such as *Helicobacter pylori* (Figure 2) [55,117]. These microbiome changes are beneficial for the host to prevent the development of inflammatory diseases and possibly cancers of the GI tract.

### 4.6. BRBs and Human Papilloma Virus Infection

As the most common sexually transmitted disease worldwide [118,119], management of HPV infection remains a significant public health concern. While most infections from HPV are cleared by the immune system in under two years of initial infection, a small fraction persists to preneoplastic lesions and result in cancers [120,121]. HPV infections are closely linked to cervical cancer but are also noted in a significant portion of anogenital and oropharyngeal cancer [122,123,124]. In cervical cancer, the fourth deadliest cancer in women, HPV infection has been found in over 90% of cases [125,126]. With the advent of multiple HPV vaccines becoming available, the incidence of cervical cancer, neoplasia, and genital warts has markedly reduced [127,128,129]. It is of interest that BRB phytochemicals display anti-proliferative activity against human cervical cancer cell lines [130]. Antiviral activity of BRB constituents and phytochemicals have been demonstrated in influenza A, Aichi viruses, and norovirus [131,132,133,134,135]. BRBs are currently being tested in a clinical study investigating the potential for application of BRB and lactoglobulin in the treatment of HPV and prevention of cervical cancer. In the test group, patients were administered SanuGene Vaginal Gel, composed of BRB extract, lactoglobulin, and other components within a gel matrix. With a sample of 300 outpatients divided into 200 tests and 100 control subjects, participants were given a vaginal preparation of gel every other day containing either 3 g BRB extract (test group) or placebo (control group). Preliminary results from the Tianjin Medical University report that within the test group, 72.8% of subjects had an effective clearance of HPV infection, whereas only 15.8% of subjects in the control group cleared their infection. Additionally, the HPV viral load within the test group decreased by 60%, whereas the control group increased by 21.8%. No serious side effects were reported. BRB extract in combinatorial treatment approaches, including vaginal gels, has strong implications and potential for the treatment of HPV infection and prevention of cervical cancers [136].

## 5. Berry Phytochemicals in Cancer Chemoprevention

### 5.1. Anthocyanins

**Figure 3:**
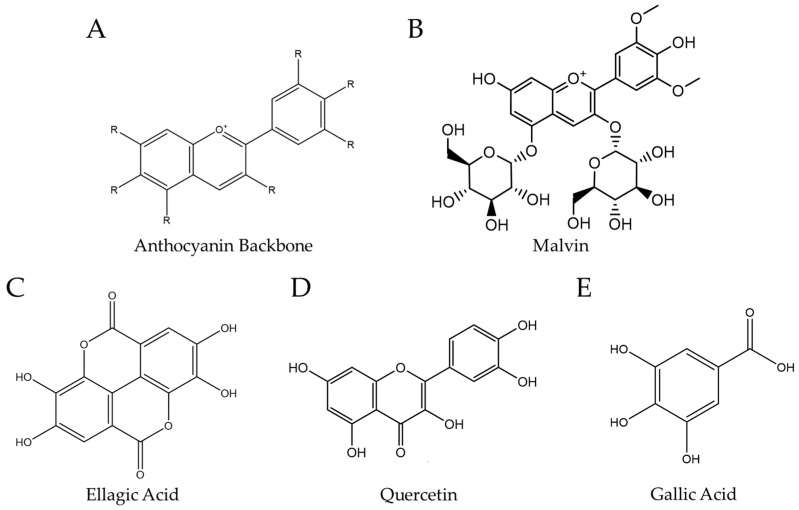
Chemical structure of (**A**) anthocyanin backbone and (**B**) a representative anthocyanin Malvin. Chemical structure of (**C**) ellagic acid, (**D**) quercetin, and (**E**) gallic acid.

Anthocyanins are the predominant class of polyphenols found in BRB [137] (Figure 3A,B). BRBs present a significantly higher concentration (669 mg/100 g) and diversity of anthocyanins compared to other berry fruits, such as blueberries (32–407 mg/100 g), strawberries, grapes, and raspberries (27–48 mg/100 g) fresh weight [137,138,139,140]. In vitro and in vivo studies have demonstrated that berries and their bioactive components exert therapeutic and preventive effects against colon cancer by the suppression of inflammation, oxidative stress, proliferation, and angiogenesis through the modulation of multiple signaling pathways such as NF-κB, Wnt/β-catenin, PI3K/AKT/PKB/mTOR, and ERK/MAPK [141,142]. Jing et al. observed that anthocyanin extracted from bilberries at 500 μMinduced mitochondrial damage and increased casp-3 and casp-9 activation in colon cancer cells [143]. This study also indicated that glycosylated anthocyanins could potentiate anticancer effects by regulating energy transport, increasing the NAD+/NADH ratio to increase mitochondrial damage by blocking energy metabolism. Meta analysis of non-randomized trials of cohort and case–control studies performed by Wang et al. indicate an inverse relationship between anthocyanin consumption and CRC risk through analysis of seven studies measuring anthocyanin consumption [144]. Effects of anthocyanins have also been tested in prostate cancers [145], which contribute to increased mortality rates in men globally [145]. Anthocyanins, and especially cyanidin-3-O-glucoside, have demonstrable anti-cancer activity against prostate cancer cells in vitro. Treatment of DU-145 prostate cancer cells with anthocyanins increased apoptosis via p53 and Bcl2 pathways [146]. Studies on various anthocyanin monomers such as Pt3G of the lyceum ruthencium plant further corroborates increased apoptosis and cell cycle arrest of DU-145 cells when treated with anthocyanin [147]. Similar mechanisms targeting Bcl2 and Bax, upregulating caspase-3 pro-apoptotic gene and increasing ROS to further induce apoptosis, were noted, indicating a method to combat the proliferation of prostate cancer [147]. Other studies have also purified anthocyanins from natural sources, such as strawberries and other fruits, to treat prostate cancer cells lines DU145 and LNCaP, demonstrating reduced cell viability to 45 and 40%, respectively, upon 48 h treatment with 100 μg/mL cyanidin 3-glucodside [148].

### 5.2. Ellagic Acid

Ellagic acid (EA), a phenolic compound present in BRBs, exhibits potential cancer chemopreventive properties (Figure 3C). The concentration of total ellagic acid in BRBs is approximately 234.2–258.4 mg/100 g, which is notably higher compared to other fruits like strawberry (25.0–78.5 mg/100 g), blackberry (30.0–54.7 mg/100 g), and blueberry (0.8–6.7 mg/100 g) [149,150,151,152,153]. EA has demonstrated anti-cancer effects in cancers such as colon cancer. Studies indicate its effectiveness in reducing CRC proliferation, inducing apoptosis, and modulating long non-coding RNAs in vitro [154]. Other mechanisms associated with EA-mediated colon cancer cell inhibition includes increased G0/G1 cell cycle arrest, with the Smad/TGF-B1 pathway crucial in promoting apoptosis and cell cycle arrest. Other studies confirm that EA induces apoptosis and autophagy in colon cancer cells and reduce tumor sizes in nude mice [155]. Additionally, recent studies have found EA to be useful in reducing toxicities associated with chemotherapy treatment of colon cancers. Administration of EA (10 mg/kg bwt po daily for 6 weeks) significantly diminished the toxicity caused by cisplatin (5 mg/kg bwt ip once a week for 4 weeks), restoring liver marker enzyme activity and reducing the prooxidative damage of the cisplatin treatment. These findings point to the potential of EA to be used not only as a dietary supplement in reducing colon carcinogenesis but also to revert peroxidative toxicities of chemotherapies when used in combinatorial treatment strategies. EA has been implicated in targeting MDM2, which downregulates the action of the p53 tumor suppressor, and treatment could reduce MDM2 and reinduce apoptosis in human prostate cancer cells [156]. Eskra et al. observed that EA and its metabolite urolithin A could reduce prostate cancer cell growth [157]. It is interesting to note that EA extracts from BRBs tested on castrate-resistant prostate cancer (CRPC) cells, potentially interferes with the effects of taxane therapy via effects on microtubule polymerization [158]. These results highlight the need for further research to understand how the antioxidative effects of EA may affect prooxidative chemotherapy regimens, and how strategic combinations with berry phytochemicals can be leveraged for prostate cancer treatment. Other challenges associated with the use of EA in prostate cancer are related to reduced bioavailability of EA, potentially due to the increased number of hydroxyl groups. This was further demonstrated in a study using pomegranate juice (which is rich in EA) in prostate cancer patients, where only 8 of 33 patients had detectable EA in their prostate tissue [159]. The chemopreventive effects of EA has also been investigated in pancreatic cancer, which is one of the leading causes of cancer death worldwide, with a global burden which has more than doubled over the past 25 years [160]. The most common type is pancreatic ductal adenocarcinoma, which is derived from cells that line the ducts that carry digestive enzymes out of the pancreas [160]. EA has been tested against pancreatic cancer cell lines, with demonstrable efficacy associated with modulation of casp-3 and casp-9 genes, as well as downregulation of TGF-B and MMP2 and MMP9 [161]. In vivo, EA has notably demonstrated anticarcinogenic properties in pancreatic tumor-bearing mice, reversing epithelial to mesenchymal transition, and restoring cell cycle arrest [162]. EA was also found to display chemopreventive efficacy in a PANC-1 xenograft model on nude mice, reducing tumor growth along with suppressing anti-apoptotic, angiogenic (COX-2, HIF1α, VEGF, VEGFR, IL-6 and IL-8), and metastatic biomarkers, as well as reducing epithelial to mesenchymal transition [163].

### 5.3. Quercetin

Quercetin (3,3′,4′,5,7-pentahydroxyflavone) is a member of the flavonoid family of compounds (Figure 3D). It belongs to the flavonoid family and is a naturally existing compound within the human diet and is present in BRB at a concentration of 100–190 mg/kg [149]. This concentration is lower than in other berries like whortleberry (158 mg/kg), lingonberry (145 mg/kg), cranberry (121 mg/kg), and blueberry (99.9 mg/kg) [164,165]. Due to its potential in preventing or treating various diseases, including cancer, commercial dietary supplements have included quercetin. Doses of quercetin between 1 and 40 μM do not appear to induce any discernible toxic side effects in normal cells, and an increasing number of researchers are focusing on its potential therapeutic impact on cancers [166,167]. Several studies have confirmed that quercetin provides various biological functions, including anti-inflammatory, antioxidant, and anti-cancer effects [167]. In vivo experiments on mice bearing CT-26 and MCF-7 tumors that were treated with 100 and 200 mg/kg concentrations of quercetin demonstrated that the survival rate was significantly higher compared to the control group [168]. Furthermore, its anti-cancer properties in OSCC were demonstrated by inhibiting cell migration through the suppression of epithelial–mesenchymal transition (EMT) and matrix metalloproteinase (MMP) in OSCC cells. Quercetin inhibits the survival and metastatic capabilities of OSCC cells by targeting the EMT pathway [169]. Zhang et al. investigated the efficacy of quercetin in the chemoprevention of chemically induced carcinogenesis in a hamster model using DMBA. They found that quercetin, administered at various doses, led to a significant decrease in the incidence of OSCC and reduced the severity of hyperplasia and dysplasia when compared to the DMBA group. Moreover, high doses of quercetin resulted in a substantial suppression of gene and protein expression of NF-ĸB p50, p65, and Bcl-2. On the other hand, medium and high doses of quercetin increased gene and protein expression of the apoptosis regulator Bax. Additionally, quercetin effectively mitigated bodyweight loss compared to the DMBA-induced group only, suggesting its potential as a candidate for preventing OSCC [170]. In a prostate cancer study, carcinogenesis which is marked by heightened activation of the IGF-1/IGF-1R axis through the PI3K/Akt or RAF/MEK/ERK signaling pathways and the expression of the androgen receptor (AR), was reduced after quercetin treatment. Quercetin was also shown to restore antioxidant enzymes and apoptotic proteins levels. The study suggests that quercetin has the capacity to inhibit the expression of proteins linked to cell survival, proliferation, and anti-apoptotic functions, ultimately contributing to the prevention of prostate cancer [171].

### 5.4. Gallic Acid

According to some estimates, BRBs contain about 965.6 ± 2.9 mg of gallic acid equivalents (GAE) per gram compared to red raspberry which is 239.60–372.0 mg/100 gm dry weight and blackberry which is 226.0 mg/100 gm [172] (Figure 3E) [173]. GA displays significant anti-inflammatory properties. Interleukin-8 (IL-8), which is a survival factor for cancer cells, and is associated with cancer metastasis, increased proliferation, and angiogenesis [174,175], was reduced by GA, resulting in a significant reduction in the proliferation of HCC cells [176]. In vivo, GA’s potential for preventing prostate cancer was assessed in a TRAMP mouse model. Mice receiving 0.3% and 1% GA presented an increased occurrence of well-differentiated lower-grade prostatic tumors, and a notable decrease in poorly differentiated tumors. GA also reduced the tumor proliferation in treated mice, and significantly inhibited the expression of proteins related to cell cycle regulation (Cdks and cyclins) in prostate tissue [177]. This study revealed that oral GA administration effectively inhibits the growth and progression of prostate cancer in TRAMP mice. In another study investigating the potential anti-cancer properties of GA in human prostate cancer cells, DU145 and 22Rv1 revealed a dose-dependent reduction in cell viability via the promotion of apoptosis. In in vivo tests using nude mice, GA reduced the growth of prostate cancer xenografts and exhibited anti-proliferative, apoptotic, and anti-angiogenic effects within tumor tissues [178].

## 6. Discussion

Preclinical and clinical studies demonstrate that BRB and its bioactive phytochemicals have significant beneficial effects linked to cancer inhibition in several cancer types along with benefits in other disease conditions. As shown in this review, published works demonstrate that BRB and its phytochemicals possess a multitude of cancer chemopreventive properties including anti-proliferative effects, anti-inflammatory abilities, activation of pro-apoptotic pathways, modulation of the microbiome, antioxidant, and immunomodulatory properties (Figure 2). These effects are summarized in Table 1. Many studies also report on the safety profile of BRB formulations in clinical studies, with minimal side effects or adverse effects reported. This serves as an added advantage over conventional chemotherapeutics as it holds the potential for integration into, or substitution for, BRB within the framework of present cancer treatment protocols. The pharmacokinetics and bioavailability of phytochemicals in BRBs has been associated with potential divergent outcomes across different study populations and groups. Overcoming this major hurdle is essential to fully exploit the chemopreventive properties of BRB bioactive phytochemicals.

Additional research is imperative to investigate methodological considerations and the potential benefits of combinatorial approaches with BRB or BRB phytochemicals in cancer chemoprevention and treatment in preclinical and clinical studies. Several review papers in the past have commented on the need for the field to begin moving towards combining natural compounds with current cancer therapies [13,36,149]. Given the wide range of anti-cancer properties, future work should include assessments of how BRBs may potentiate the cancer inhibitory properties of chemotherapy, radiation therapy, immunotherapies, and other treatment modalities. These combined studies may reveal potential enhancements in efficacy of treatments compared to mono-therapy approaches, which will benefit patients. As of 2023, there are now several ongoing clinical trials investigating BRB combinational treatments with chemotherapy, immunotherapy, or radiotherapy. Mounting evidence for the potential of BRBs to improve cancer patient treatment outcomes cannot be understated. As more preclinical evidence emerges, findings supporting BRB administration in clinical settings are becoming more abundant. Advancements in treatment options for patients occur frequently and the potential of combinatorial approaches with a BRB diet could improve patient outcomes. Some clinical trials within the recruitment stages are beginning to investigate the effects of dietary intervention or changes in combination with immunotherapy (NCT05356182, NCT04645680, NCT04866810, NCT05832606).

In this review, we have also explored the health benefits that BRBs possess for other diseases and conditions. It is important to recognize the potential that BRB administration can have on the treatment of other diseases and conditions, as it can further reveal novel therapeutic properties BRBs possess that could benefit the treatment of various cancers. Furthermore, the cancer research properties discussed in this review may provide novel ways to treat other diseases and conditions. An example of this comes from the microbiome modulatory properties of BRBs, as discussed in this review. Major alterations in the microbiome of the gut and oral cavity have been associated with the development and progression of several diseases like inflammatory bowel disease and irritable bowel syndrome, metabolic diseases like obesity and diabetes, allergic conditions, and neurodevelopmental illnesses, along with several cancers including the ones discussed in this review [102,103,104,179,180]. Understanding how the composition and behavior of the host microbiome is modulated during BRB administration can potentially provide mechanistic evidence underlying BRB-mediated suppression of cancer development and treatment of other disease conditions.

Natural compound drug discovery provides a unique area for collaboration between academia and industry; however, there are challenges associated with a focused effort to support such translational research [181]. Other challenges arise due to the variability in natural compound composition in plant material due to factors like variation in locations where the plants are grown and collected [182]. Furthermore, identifying, isolating, and characterizing bioactive compounds can also be a significant challenge along with the generation of structural analogues if the synthetic routes are problematic [183,184]. Analytical techniques, genome mining and engineering, and cultivation systems are among the current technological and scientific advances that may overcome these challenges [181].

This review has discussed several methods by which researchers have introduced BRBs into animal models and human patients. Variation within individuals of the same group have been reported; thus, further studies in the pharmacokinetics of different BRB administration routes and protocols are required. The route and method of BRB administration will likely vary with different cancers and diseases. Dosage, duration, and mode of BRB delivery for cancer treatment is essential to achieve optimal outcomes, and this has been addressed in other reviews [13,36,149]. Further study of these methods of delivery may prove useful in translating BRBs and natural compounds to clinical study for improved results.

## 7. Final Remarks

It is our opinion that to advance BRB phytochemical chemoprevention studies, preclinical and clinical research should focus on (1) assessments of BRB combinational therapies with existing cancer treatments; (2) optimization of BRB delivery systems and improvement of bioactive phytochemical pharmacokinetic properties; and (3) further investigation of BRBs’ impact on metabolism and modulation of microbiome. As basic research continues to inform on the vast opportunities that BRB-mediated chemoprevention provides for patients undergoing cancer treatment, the need to continue to support BRB chemoprevention clinical trials cannot be understated. Furthermore, the utilization of BRB phytochemical treatment should be further expanded into other diseases, and conditions, as its health benefits have continued to show promising results.

## Figures and Tables

**Table 1:** Impact of BRB in various cancers: insights from in vivo, in vitro, and clinical studies.

Cancer	Study	Model/Patients	Methods and Doses	Results	Ref
**Head and neck cancer**	In vivo	DMBA-HNSCC model; HCPs	Topical and dietary 5% and 10% BRB; 6 weeks	Reduced tumor multiplicity, incidence, and proliferation rate	[23]
		4NQO-HNSCC model; F344 rats	Dietary 5% and 10% BRB; 6 weeks	Reduced level of Aurka, Birc5, Ccna1, Ccna 2 and ki67	[25]
		4NQO-HNSCC model; C57BL/6 mice and F344 rats	Dietary 5% and 10% BRB; 25 weeks	Increased expression of HSD11B2, which converts cortisol to cortisone	[26]
		4NQO-HNSCC model; C57BL/6 mice	Ethanol BRB extract, 24 weeks	Inhibits recruitment of regulatory T cells, enhance CD8+ T-cell activity in TME	[12]
	In vitro	CAL27 cells	BRB extract at 20 and 200 μg/mL; 24 h	Both doses reduced PCNA, BCL-2, and CDK1A levels, reduced cortisol level at low dose	[26]
	Clinical	HNSCC patients	Applied mucoadhesive gel of 10% BRB-FD; 6 weeks	Histological regression and reduction of loss of heterozygosity, lowered COX-2 level.	[28]
		Biopsy confirmed OSCC patients	3 oral troche/day with 4.3 g BRB-FD. 13.9 ± 1.27 days	Reduced pro-survival gene (AURKA, Birc5 and EGFR) and proinflammatory genes (NF-Kb1 and PTGS2)	[32]
**Esophageal cancer**	In vivo	NMBA rat model of ESCC in F344 rats	Dietary administration of 5% BRB; 30 weeks	Reduced tumor incidence, multiplicity, increased oxidative stress, and suppressed NFκB/MAPK signaling.	[39]
		NMBA rat model of ESCC	6.1% BRB powder, AC-rich fraction of BRBs (3.8 μmol/g); 30 weeks	Increased anti-inflammatory cytokines IL-10 and IL-12, decreased pro-inflammatory cytokine IL-1β in all diets.	[43]
	In vitro	Rat esophageal epithelial cells, RE-149 and RE-149 DHD	BRB-EtOH (10–100 μg /mL), anthocyanins (10–50 μg/mL); 4–72 h	BRB (100 µg) and anthocyanins (50 µg) induce apoptosis in RE-149 DHD, anthocyanins (50 µg) decreased cell proliferation in both cell lines	[44]
		ESCC cell; KYSE-150	Cyanidin-3-O-glucoside, cyanidin-3-O rutinoside (0–300 μM); 24–72 h	Suppressing human β-defensin 2 (HBD-2), an oncogenic protein	[45]
	Clinical	Barrett’s esophagus patients	Lyophilized BRB at 32 g and 45 g once daily; 12–26 weeks	Increased urinary excretion of 8-PGF2α (lipid peroxidation marker associated with oxidative stress)	[46,47]
**Colorectal cancer**	In vivo	AOM-CRC model in mice	7 μmol/g BRB anthocyanin equivalent to 10% BRB-FD powder; 39 weeks	Lowered SIRT-1, upregulated Bax, and CRC cell cycle arrest. Combinatorial treatment reduced tumor multiplicity, inhibition of AKT signaling pathway.	[53,73]
		AOM/DSS-CRC model in mice	3.5 or 7.0 μmol/g of BRB anthocyanins. 5% BRB diet; 4 weeks	Anthocyanins lowered IL-1β, IL-6, COX2 and TNF-α, suppressed adenocarcinoma progression; increased NK cells at tumor site	[55,58]
	In vitro	SW480, Caco2 cells	BRB anthocyanin (25 μg/mL); 24 h	Increase histone acetylation through reduction of SIRT1, upregulation of p65, NF-kB, Bax and inhibition of Bcl-2, cyclin-D1, c-myc and NLRP3 expression.	[53]
		SW480, Caco2 cells	BRB-Ant (50 μg/mL); SW480 (5-FU: 32 μM, Cel: 60 μM), Caco2 (5-FU: 50 μm, Cel: 60 μM)	BRB with 5-FU or Cel inhibited CRC cell proliferation; revered expression of drug resistance gene, MDR1.	[73]
	Clinical	Colon biopsy, 9 patients	BRB consumption; 4 weeks	Higher tumor-infiltrating NK cells (CD56+), NK cell cytotoxicity (CD56+CD107a+)	[58]
		Phase-I, 24 patients	20 g BRB-FD; thrice/day; 1–9 weeks	BRB promoted antiproliferative, anti-angiogenic effect, discouraged CRC progression, with IL-8 and GM-CSF as prognostic markers.	[59]
		Phase-I, BRB diet	20 g BRB-FD thrice/day; 1–9 weeks	Decreased β-catenin, Ki-67, and CD105; increased expression of TUNEL and p16, supporting its anti-proliferative and antiangiogenic role.	[61]

ESCC: Esophageal squamous cell carcinoma, AOM/DSS: Azoxymethane/Dextran sodium sulphate, BRB-FD: black raspberries—freeze dried, DMBA: 7,12-dimethylbenzanthracene, NMBA: N-nitrosomethylbenzylamine, HCPs: hamster cheek pouches, TME: tumor microenvironment. In vivo studies in green, in vitro studies in blue, and clinical trials in orange.

## Data Availability

Data sharing is not applicable.
